# Enabling population assignment from cancer genomes with SNP2pop

**DOI:** 10.1038/s41598-020-61854-x

**Published:** 2020-03-16

**Authors:** Qingyao Huang, Michael Baudis

**Affiliations:** 1 Institute of Molecular Life Science, University of Zurich, Winterthurerstrasse 190, 8057 Zurich, Switzerland; 2 SIB, Swiss Institute of Bioinformatics, Winterthurerstrasse 190, 8057 Zurich, Switzerland

**Keywords:** Cancer genomics, Data mining, Epidemiology

## Abstract

In many cancers, incidence, treatment efficacy and overall prognosis vary between geographic populations. Studies disentangling the contributing factors may help in both understanding cancer biology and tailoring therapeutic interventions. Ancestry estimation in such studies should preferably be driven by genomic data, due to frequently missing or erroneous self-reported or inferred metadata. While respective algorithms have been demonstrated for baseline genomes, such a strategy has not been shown for cancer genomes carrying a substantial somatic mutation load. We have developed a bioinformatics tool for the assignment of population groups from genome profiling data for both unaltered and cancer genomes. Despite extensive somatic mutations in the cancer genomes, consistency between germline and cancer data reached of 97% and 92% for assignment into 5 and 26 ancestral groups, respectively. Comparison with self-reported meta-data estimated a matching rate between 88–92%, mostly limited by interpretation of self-reported ethnicity labels compared to the standardized mapping output. Our SNP2pop application allows to assess population information from SNP arrays as well as sequencing platforms and to estimate the population structure in cancer genomics projects, to facilitate research into the interplay between ethnicity-related genetic background, environmental factors and somatic mutation patterns in cancer biology.

## Introduction

Cancer arises from the accumulation of genomic aberrations in dividing cells of virtually all types of proliferating tissues (somatic variations). The irregular cellular expansion and other hallmarks of cancer can result from a plethora of mechanisms affecting multiple cellular processes^1^. Some oncogenetic pathways can be initiated by exogenous factors, e.g. tobacco smoke or ultraviolet radiation^2^. However, exposure to carcinogenics varies in its effects for people from different genetic background, which suggests that somatic mutations can be influenced by inherited (“germline”) variations^3,4^.

Cancer studies have reported significant world-wide variation in incidence and prognosis between ethnicity groups^5–8^. While such differences have been attributed to unequal social and economical circumstances which influence risk factors and therapeutic interventions, several studies have shown impact of population specific genomic variants with predisposing effects on malignant transformation and phenotypic behaviour^9–12^. Due to the late onset of most cancers, even high-penetrance Mendelian-type variants may not be purged by natural selection and can accumulate in particular populations. Such variants may play key roles in cancer development^13^. Notably, mutations on BRCA1/2 genes confer a high risk to develop breast and ovarian carcinomas. Three founder mutations in Ashkenazi Jewish population cause the BRCA1/2 mutation prevalence to be 10-fold higher than all sporadic mutations in the general population^14,15^. Mitochondrial aldehyde dehydrogenase (ALDH2) encodes an enzyme in alcohol metabolism. One variant, ALDH2*504Lys, which increases risk for alcohol-related liver, colorectal and esophageal cancer by alcohol consumption, has 36% prevalence in East Asian populations^16,17^.

Many other studies have reported prevalent genetic variants in specific population groups which may contribute to the observed disparities in occurrence and prognosis among populations^18–20^. As patients of European ancestry are overrepresented in medical research and clinical studies, we have a biased view on genetic aetiology in diseases and available therapeutic approaches may not benefit patients with other genetic backgrounds. Studying less explored ancestral backgrounds may lead to discovery of new biological mechanisms and therapeutic prospects. Although high-impact monogenic germline variants are rarely expected in cancer, polygenic variation models for breast cancer which estimate the combined risk profile of multiple loci have been developed, suggesting the potential of exploring genome-wide risk profiles^21^. Another study on germline background of cancer genomes has also identified disease-associated chromosomal regions from only seven individual samples with linkage mapping^22^. This type of studies can be conducted population-wise, with sufficient number of samples from the same population or ethnicity group.

With the increasing number of available genome profiles and the decreasing cost to genotype clinical samples, the stratification between patients’ genetic backgrounds has become feasible with the promise to guide therapeutic strategies and improve the clinical prognoses. Since several studies have demonstrated the relevance of considering an individual’s genomic origin for preventive screening (reviewed in Foulkes *et al*.^15^), information about the population background of cancer patients may be an additional factor for individual therapeutic decisions as well as for the stratification of clinical study cohorts. A meta-analysis addressing the interplay between genetic background, cancer development and therapeutic responses is desirable, not only for robust statistical associations in molecular target identification, but also for the rational design of studies incorporating informative biosamples.

For many data repositories, “population group” of a sample can be assumed based on a geographical location associated with the sample. Alternatively, a self-reported “race” category is commonly used in the U.S. census data^23,24^. A biosample’s geographic origin is often approximated using the location of the study’s research facility or the contact address of its main authors^25^. However, while these data can be easily retrieved, they may not provide an accurate representation of patients’ origins for the purpose of population-specific ancestry mapping. Self-reported data are often inconsistent across studies, vague in category description (e.g. *“white”*, *“black”* v.s. *“Caucasian”*, *“African”*) and misleading when patients have incomplete awareness of the migration and admixture histories of their ancestors. Overall, when associating oncogenic molecular signatures with germline variations, information from the above sources lacks in relevant detail and consistency.

**Figure 1:**
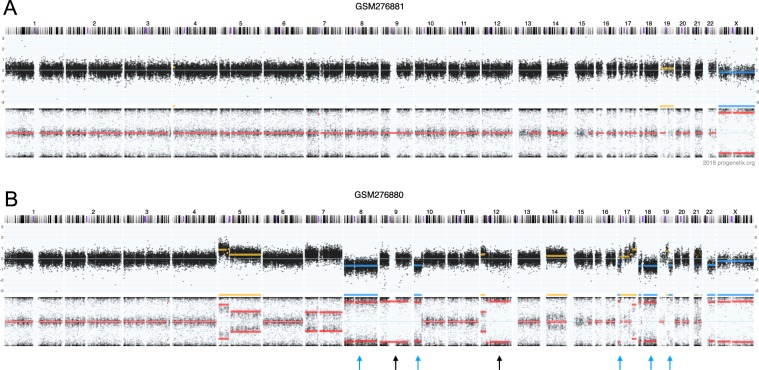
CNV tracks for a paired normal/cancer samples deposited in GEO with sample IDs GSM276881 and GSM276880. GSM276880 is a glioblastoma sample. GSM276881 is its peripheral blood control. The CNV tracks are consisted of two panels. Upper panel indicates the total copy number, represented by logR ratio ($lo{g}_{2}\frac{copy\,number}{reference\,copy\,number}$ at any probe) Namely, logR = 0 indicates a position with normal two copies, logR > 0 indicates a copy number gain and logR < 0 indicates a copy number loss. Lower panel indicates the allele specific copy number, represented by B-allele frequency (BAF, $\frac{No.\,B\,allele\,copy}{Total\,copy\,number}$). Any given SNP position can have a value between 0 and 1. A line at 0.5 indicates a heterozygous region. Compared to the unaltered genome (A), the cancerous counterpart (lower) has copy number loss in chr8, 10p, 18, 19qter, 22q and copy-neutral loss of heterozygocity in chr9,12q, accounting for 18.2% of genome.

A better approach to population assessment would be the computational estimate of ancestry with population-specific genomic variants. This has been shown previously for germline profiles, achieving 90% accuracy to distinguish three populations, African American, Asian and Caucasian, by using as few as 100 population-diverging single nucleotide polymorphisms (SNPs)^26^, and nowadays is a standard methodology with claimed better granularity behind a number of commercial “ancestry” services, e.g. 23andme.com, myheritage.com and ancestry.com. We hypothesise that a similar strategy can be applied to cancer genome data, despite the additional cancer-related somatic mutations which leads to both information loss (e.g. large scale homozygous or allelic deletions) and added noise (e.g. somatic mutations masking germline variants). An example of a cancer genome containing copy number loss and copy-neutral loss of heterozygocity (CN-LOH) events and its paired normal sample is shown in Fig. 1. It is a typical case of altered genome with copy number loss (blue arrow) with CN loss regions partly recovered by doubling of the second allele (CN-LOH, black arrow) at multiple genomic locations. In addition to a general test of feasibility of inferring population background from the noisy cancer genome data, we continue with benchmarking population mapping procedures for heterogeneous datasets from different genotyping platforms, with the aim of integrating cross-platform cancer genotyping data for meta-analysis.

## Results

We developed SNP2pop pipeline to assign genomic data to ancestral groups defined by 1000 Genomes Project^27^, i.e. five continental groups and 26 population groups, respectively: 1. Admixed American (AMR) includes: Colombians from Medellin, Colombia (CLM), Mexican Ancestry from Los Angeles USA (MXL), Peruvians from Lima, Peru (PEL) and Puerto Ricans from Puerto Rico (PUR); 2. African (AFR) includes: African Caribbeans in Barbados (ACB), Americans of African Ancestry in SW USA (ASW), Esan in Nigeria (ESN), Gambian in Western Divisions in the Gambia (GWD), Luhya in Webuye, Kenya (LWK), Mende in Sierra Leone (MSL), and Yoruba in Ibadan, Nigeria (YRI). 3. East Asian(EAS) includes: Chinese Dai in Xishuangbanna, China (CDX), Han Chinese in Beijing, China (CHB), Southern Han Chinese (CHS), Japanese in Tokyo, Japan (JPT) and Kinh in Ho Chi Minh City, Vietnam (KHV); 4. European (EUR) includes Utah Residents with Northern and Western European Ancestry (CEU), Finnish in Finland (FIN), British in England and Scotland (GBR), Iberian Population in Spain (IBS) and Toscani in Italia (TSI); 5. South Asian (SAS) includes: Bengali from Bangladesh (BEB), Gujarati Indian from Houston, Texas (GIH), Indian Telugu from the UK (ITU), Punjabi from Lahore, Pakistan (PJL) and Sri Lankan Tamil from the UK (STU).

We benchmarked this tool with various normal and cancer samples from independent datasets to demonstrate the feasibility and reliability of this approach.

### Cross-platform benchmarking

**Figure 2:**
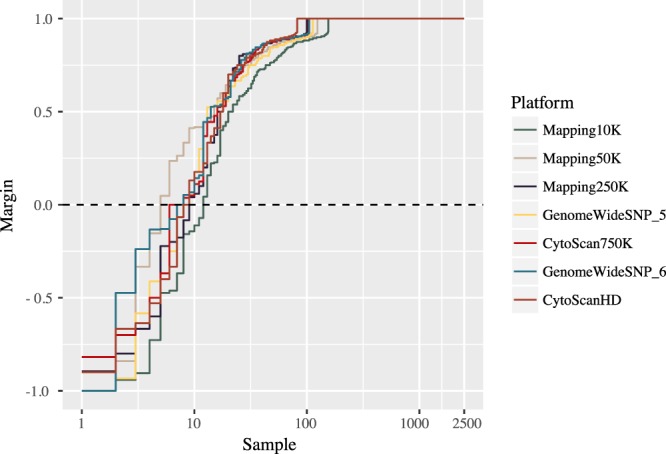
Estimating the model accuracy with reference samples with SNPs extracted from genotyping platforms of seven different resolution levels. Here we use margin as an estimator for the accuracy of the prediction model with random forest classifier output. It is defined as difference between the proportion of votes for the correct class minus maximum proportion of votes for the other classes. A positive value indicates correct prediction. For 2504 samples in the 1000 Genome Project, the number of incorrectly predicted samples ranged from 4 to 11, corresponding to an error rate of 0.16% to 0.4%. Note that this is cross validation error on reference dataset, rather than real data.

We first used the original data from 1000 Genomes to validate the level of resolution needed for accurate population assignment from the pipeline. Taking the sequencing data of 2504 samples and extracting the SNPs from the nine genotyping platforms of interest gave rise to the dataset in this benchmarking experiment. The number of SNPs per platform ranged from 10 204 (Affymetrix Mapping 10 K) to 934 946 SNPs (Affymetrix Genome Wide SNP 6). For all nine genotyping platforms (of seven levels of resolution), the model performed equally well in capturing the informative SNPs and predicting the population category. The assignment of 2504 samples from 1000 Genomes Project into 5 continental groups had low margins of error for all genotyping platforms (Fig. 2). To benchmark the separation of 26 groups, we applied a repeated cross validation (CV) on both random forest and multinomial linear regression model for the assignment task. Here, the CV accuracy rate ranged between 75–85% depending on platform resolution. When combining pairs of frequently mixed groups into 10 categories, a CV accuracy rate rose to >99% (Fig. S3 and Table S3).

### Benchmarking normal genome profile assignment with HapMap data

To validate the accuracy of our tool to map population origins from non-cancer SNP datasets, we used 112 samples from the HapMap project^28^ which is not included in the reference set, but acquired through GEO. After a further examination for family relatedness, we excluded 67 first-degree relatives to samples from 1000 Genomes project. By HapMap metadata, the 45 samples came from three distinct population groups (at the level of 26 population groups, from three different super population groups): CEU, CHB and YRI. Except for one sample with “CEU” label predicted as “AMR”, the assignment of the rest of samples all matched their ethnicity information indicated in the meta-data (Table 1).

**Table 1:** Comparison of HapMap metadata and predicted population group. Each column indicates HapMap population labels. Each row indicates the label predicted by the tool. CEU stands for Utah residents with Northern and Western European ancestry from the CEPH collection, CHB stands for Han Chinese in Beijing, China. YRI stands for Yoruba in Ibadan, Nigeria; AFR stands for African; AMR stands for Admixed American; EAS stands for East Asian; EUR stands for European.

	CEU	CHB	YRI
AFR	0	0	14
AMR	1	0	0
EAS	0	6	0
EUR	24	0	0

### Paired cancer-normal comparison

One of the highlights in this tool lies in the determination of population origin from cancer genome profiles carrying various acquired genomic aberrations. Since the non-cancer samples could be correctly assigned according to HapMap categories, we then validated the cancer genome based assignments in samples where normal genome profiles of the same patients (e.g. from peripheral blood or non-cancer tissue samples) were available as reference. We performed the validation with two independent data sources — GEO and TCGA project.

#### GEO data

**Figure 3:**
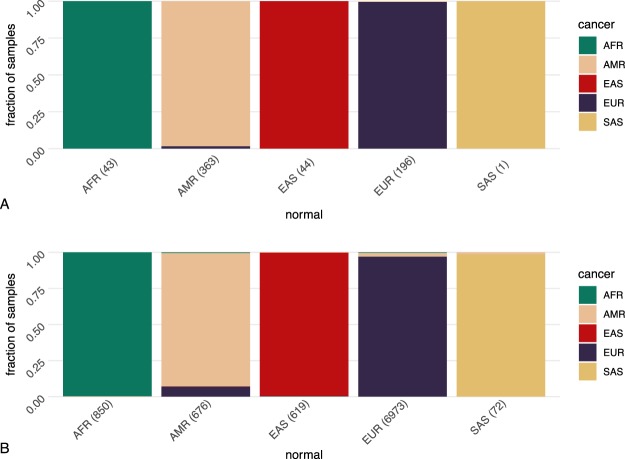
Accuracy of assignment with paired tumor and normal samples from GEO and TCGA project. 647 (score > 0.2) individuals from GEO (**A**) and 8216 (score > 0.2) individuals from TCGA project (**B**) with paired tumor and normal samples with paired tumor and normal samples were examined. The columns are the prediction groups for the normal samples and the color codes indicate the proportion of each group predicted from their respective cancer samples.

From the GEO repository, we retrieved all paired normal and cancer samples from 1145 individuals and compared the outcome of the population assignment. When including all 1145 individuals, 92.1% of the normal samples matched with paired tumor samples. With an increasing confidence score, the matching accuracy increased (Table S4). The error rate dropped from 15.9% to 1.5 % comparing samples with score range “0.2–0.4” to those with “0–0.2”. After setting a threshold of normal samples with score >0.2, 95.8% accuracy could be achieved for the remaining 688 individuals. When also setting the score threshold for cancer samples to >0.2, 98.9% of the 647 remaining samples could be matched correctly (Fig. 3A). This comparison suggested a high accuracy rate in population assignment for cancer samples and an increase in the level of accuracy with a lower admixture background of the individual.

#### TCGA data

We used the genomic array profiling data from TCGA project, all of which originate from the array platform Genome Wide SNP 6. We extracted all 18380 samples (of 9190 individuals), where a normal tissue control for the respective tumor sample was available. 8924 out of 9190 (97.1%) individuals had matched tumor/normal categories. After setting a threshold of normal samples with score >0.2, 98.4% accuracy (8413 out of 8549 individuals) can be achieved. When also setting the score threshold for cancer samples to >0.2, 8195 of the 8216 (99.7%) remaining samples could be matched correctly (Fig. 3B). The score and error relation was depicted in the Table S5.

For population assignment into 26 groups, 8522 out of 9190 (92.7%) pairs had matching categories (Fig. S4). With the same score thresholding as described above, the accuracy increased to 97% (7374 out of 7602) and 99.1% (6948 out of 7010) respectively.

#### Modification of SNP status during carcinogenesis

We further scrutinized the source of noise and information loss during carcinogenesis. Using the same dataset, we performed a SNP-by-SNP comparison between the paired normal and cancer samples and summarized the SNPs which changed from heterozygous to homozygous (Het2Homo), from homozygous to heterozygous (Homo2Het) or stayed identical (Fig. S5). The Het2Homo (6.3% on average) transition occurred in case of allele loss or copy-neutral loss of heterozygocity and constituted the larger part to noise in cancer samples. The Homo2Het (5.6% on average) transition could come from loss of both alleles causing BAF to appear at 0.5 or less frequently when somatic point mutations coincide with germline polymorphism sites. In this section, We benchmarked the cancer/normal assignment consistency with presence of these two types of SNP status modifications. We also indirectly addressed the issue of tumor sample purity here, as mixed cancer/normal samples would be assigned to same categories as either of the pure cell populations.

### Comparison with self-reported ethnicity metadata

To assess the overall accuracy of self-reported population information, we compared the meta-data from GEO and TCGA with our benchmarked results.

#### GEO data

**Figure 4:**
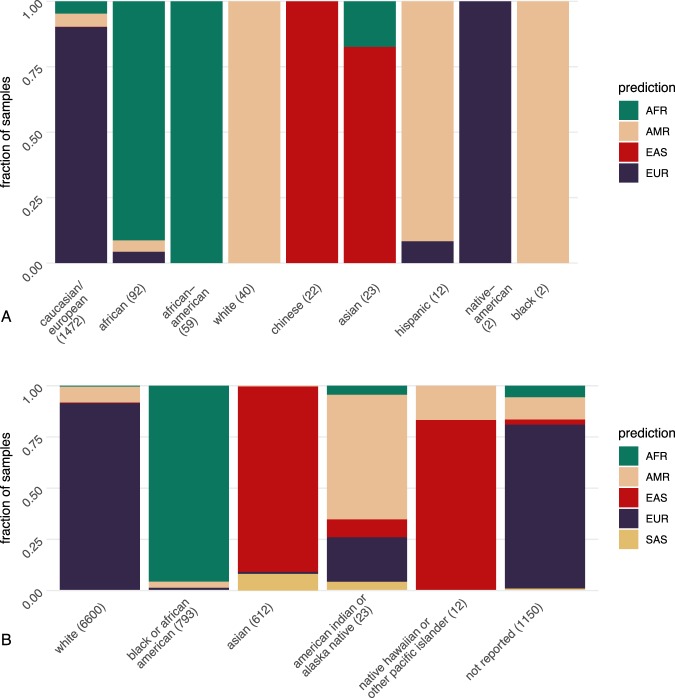
Accuracy of assignment with self-report metadata from GEO and TCGA project. (**A**) The composition of assigned super population groups of 1724 samples from GEO with extracted population/ethnicity metadata in nine groups: “african”(92), “african-american”(59), “black”(6), “caucasian/european”(1472), “white”(40), “asian”(23), “chinese”(12), “hispanic”(12) and “native american”(2). (**B**) The composition of assigned super population groups of 9190 samples in six “race” categories from TCGA meta-data: “American Indian or Alaska native”(23), “Asian”(612), “Black or African American”(793), “Native Hawaiian or other Pacific Islander”(12), “White”(6600) or “Not Reported”(1150).

We retrieved a total of 1724 samples with intelligible self-reported metadata from GEO. We extracted the population-implying keywords, which formed nine groups: “african”(92), “african-american”(59), “black”(6), “caucasian/european”(1472), “white”(40), “asian”(23), “chinese”(12), “hispanic”(12) and “native american”(2) (Fig. 4). Specifically, “african” and “african-american” samples were mostly assigned to “AFR” group (91.3% and 93.6% respectively). The 1472 “caucasian/european”-labeled samples were assigned to “EUR” (90.2%) with small fraction assigned to “AFR”(4.6%) or “AMR”(5.0%). 11 out of 12 “hispanic” samples were assigned to “AMR” with one as “EUR”. All 40 “white” samples were assigned to “AMR”. These 40 samples derived from the same study, so the patients were likely from a similar ethnicity background and reported as “white” by the study. The “asian” samples were mostly assigned to “EAS” group (19/23). All 12 “chinese” samples were correctly assigned to “EAS”. Two ‘native-american’ samples were both assigned to “EUR”. Counting all samples, a meta-data matching rate of 88.3% was achieved. This indicated the existing heterogeneity in ethnicity description across studies and a certain degree of inaccuracy in self-reported ethnicity information.

#### TCGA data

From the test of paired cancer/normal samples in TCGA, we also compared our results of 9190 normal samples with the “race” attribute provided by the metadata. There, six categories were distinguished: “American Indian or Alaska native”(23), “Asian”(612), “Black or African American”(793), “Native Hawaiian or other Pacific Islander”(12), “White”(6600) or “Not Reported”(1150). The fraction of assignment results in each of these categories were shown in the bar plots in Fig. 4B). Most samples reported as “white” were assigned to “EUR”(91.4%) with 7.7% “AMR”; Most samples reported as “Black or African American” were assigned to “AFR” (95.6%) with “AMR” (3.0%) and EUR (1.0%); The “Asian”-labeled samples were composed of 90.5% “EAS” and 8.2% “SAS”. 14 out of the 23 “American Indian or Alaska Natives”-labeled samples were assigned to “AMR” and 5 to “EUR”. In the 12 “Native Hawaiian or other Pacific Islanders” samples, 10 were assigned to “EAS” and 2 to “AMR”. The samples were also assigned to one of the 26 population groups (Fig. S6), but there was no more meta-data for a comparison on this level. If we expect the following three “race” groups to match the assignment: “White” to “EUR”, “Black or African American” to “AFR” and “Asian” to “EAS” and “SAS”, then a total matching rate of 92.4% was achieved with 95.6% in African group, 98.6% in Asian group and 91.4% in European group. Despite the high matching percentage on the level of super population group in the self-report groups with large sample numbers (“White”, “Black or African American” and “Asian”), one may still argue that with the prediction outcome from our tool, the “race” information defined in the project could be well complemented and adapted for a quantitative measure for genetic background assessment.

## Discussion

The presented method resolves two issues that have been missing in the earlier approaches to estimate population structure from genotyping data: 1. it allows the integration of platforms with heterogeneous, non-overlapping SNP positions for cross-platform meta-analysis; 2. it confirms the possibility of deriving the population origin using data from aberrant cancer genomes in addition to those of unaltered genomes.

In our study, we establish the robustness of deriving population despite platform heterogeneity by cross-platform benchmarking. We show that for all the genotyping platforms used in the study, our pipeline achieves similarly high prediction accuracy, even for the lowest resolution platform with around 10 000 SNPs. This helps to integrate genotyping data generated with different platforms for meta-analysis, where the heterogeneity in resolution does not pose great impact on the parameters of interest (e.g. cancer copy number variation studies).

We demonstrate the feasibility of incorporating aberrant cancer genomes in a step-wise manner. First, we benchmark the method using samples with unaltered genomes (non-cancer) which are different from the reference dataset, and show a high matching accuracy. In addition, we simulate different levels of LOH from unaltered genomes and observed mis-assignment or decrease of confidence score only at high coverage of LOH (LOH in >60% genome) (Fig. S7). Then, we employ paired normal/cancer samples and benchmark the assignment consistency between them. With two independent data resources (GEO and TCGA), we show that the population group estimated from unaltered genome of the same individual matches that from the aberrant cancer genome with a high accuracy rate.

From the paired sample comparison, we do observe a certain degree of mismatches. These often occur in samples with a highly admixed background (indicated by lower score), rather than related to whether they are normal or cancer samples as discussed above. Furthermore, the mismatch occurs mostly between the AMR and EUR labels, which is partly expected as the 1000 Genomes Project describes “AMR” group as “Admixed American”. Clearly defining indigenous American population as reference is impractical due to the admixture events in the last hundreds of years in South America. Such admixture events inevitably pose a challenge when establishing stable reference groups for assessing the impact of population background on secondary phenotypes like mutational patterns in cancer. As many human populations are still limitedly represented or missing and major contributions to human genetic variations are emerging^29^, genomic population models will be amenable for re-assortment. Accordingly, our SNP2pop tool allows user-specified labeled reference data upload and assign population labels to the unknown samples according to the provided reference groups.

To conclude, we have developed a bioinformatics tool to assign population group based on SNP genotyping array data. We demonstrate its feasibility and accuracy on cancer samples, where somatic mutations may obfuscate part of the ancestry related SNP signal. This work can facilitate the re-analysis and meta-analysis of available cancer data by grouping samples with similar genetic background to study the potential genetic predisposition to cancer. In addition, our method provides the basis for subsequent haplotype phasing and refinement of genomic landscape for emerging somatic variation. With this tool, researchers will be able to integrate cancer genome profiling data from multiple resources to better assess the contribution of population background in population-specific mutation patterns occurring in cancer.

## Methods

**Figure 5:**
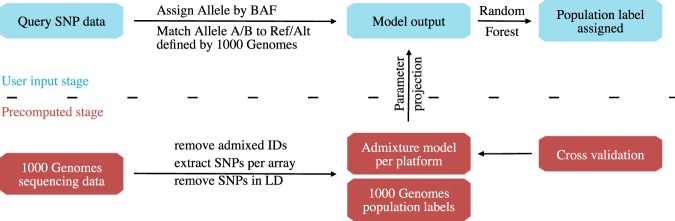
Pipeline of our tool to derive population assignment for individual cancer samples. The development workflow of the tool starts with retrieving the sequencing data from 1000 Genomes Project, removing IDs with admixture origin, extracting SNPs according to the genotyping array platforms and removing SNPs in linkage disequilibrium (LD). The generated PLINK files are stored as an intermediate reference file set. This file set is used further to train an admixture model with the model parameter matrix (allele frequency and ancestry fraction) stored for reference. Finally, a random forest classification is trained for the reference data based on their annotated population labels. These pre-computation steps are the basis of the tool, which saves time and preserves inter-study consistency. Now, when the new genotyping data comes in, the tool will assign the allele by B-allele frequency (BAF), check for the reference/alternative base for each SNP to match the reference data, assign the missing alleles (homozygous SNP from incoming data, thereby alternative base not available) based on reference SNP annotation. When the data is cleaned and compatible, the tool will use the pre-computed model parameters to analyze the admixture in the incoming dataset. Finally, with the model output, the population group of the incoming data is assigned. Users can choose to output the model parameters as well as the population groups.

We used whole genome sequencing data from the 1000 Genomes Project^27^ as reference data, which contains 2504 individuals from 26 population groups out of 5 continental groups. Allele information for SNP positions measured by the 9 selected array platforms was retrieved. In order to achieve between-study consistency for selection of informative SNPs, we used admixture model^30^ to pre-compute parameters optimized for each genotyping platform based on the reference data. The allele frequency and ancestry fraction parameters were projected to the incoming cancer dataset of the same platform. Then, by applying a random forest classification, we assigned the population label to the highest voted group and produced a score for the difference between highest and second highest percentage votes. The overview of the pipeline is shown in Fig. 5. The tool is accessible for direct use as a Docker image “https://hub.docker.com/r/baudisgroup/snp2pop” on DockerHub and its source codes can be found on Github in project “https://github.com/baudisgroup/snp2pop”.

### Data preparation

#### Reference data preparation

The SNP positions for each platform were acquired from Affymetrix annotation files. The allele information was extracted for all positions with vcftools^31^. The 12 mislabeled or admixed individuals were removed from the reference dataset, leaving 2492 individuals. The SNP positions with duplicated rsIDs in annotation files were removed. The reference or alternative strands were swapped to match the SNP array annotation. Sites with minor allele frequency (MAF) of less than 5 percent were removed. SNPs were subsequently pruned for independence using PLINK 1.9^32^. Specifically, a sliding window of 50 bp and a 5 bp shift of window at each step of pruning, with the variance inflation factor (*VIF* = 1/(1 − *R*^2^), with *R*^2^ equal to the multiple correlation coefficient for a SNP regressed on all other SNPs) at 1.5. The result files were stored as PLINK output for each platform in .bed, .bim and .fam formats, of which the .bim files were used to extract SNP positions from target data.

#### Target data preparation

The SNP array data were processed with ACNE R package^33^ to extract allele-specific copy numbers as B-allele frequencies (BAF). SNPs were labeled as homozygous A, heterozygous AB or homozygous B by the BAF value in ranges 0–0.15, 0.15–0.85 or 0.85–1, respectively, to allow both for noise and expected aneuploidy in the biosamples.

Data used for benchmarking are accessed through arrayMap database^34^, using a collection of re-processed genotyping series from the Gene Expression Omnibus (GEO) repository^35^, and the TCGA data repository^36^ from https://cancergenome.nih.gov. The samples deposited in the former were submitted by individual research studies, whereas the latter coordinated cancer genome data generation and processing with centralized protocols.

### Admixture model

While many approaches use principle component analysis (PCA) to select informative SNPs for population assignment, deriving these SNPs prior to clustering methods results in varying sets of SNPs between datasets. Here, we used the reference panel to train an admixture statistical model^30^, to derive the allele frequency in theoretical ancestor groups for each SNP. The number of theoretical ancestor groups (K) was chosen to be 9 by cross-validation. The ancestry fraction plot for reference individuals demonstrates a proper information extraction to distinguish the five continental categories. By projecting the model parameters to a new sample with the corresponding platform, a robust and consistent output with 9 ancestry fractions was generated. The ratio of the 9 fractions in each individual in the reference data of 26 population groups for each platform is shown in Fig. S1.

### Random forest label assignment

The nine ancestry fractions from the reference population were used to build a random forest model in each platform to predict the 26 population categories. The score was calculated as the difference in percentage votes between the best and the second best predicted labels. We performed repeated cross validation experiments (5-fold cross validation with 10 repetitions), and discovered twelve individuals assigned into a category different from the labels defined in their meta-data, which might occur due to a platform-specific detection of rare alleles that segregate in these samples. They were removed from reference list of the model. Finally, 2492 samples were used as training set in the classification model (removed IDs and information are found in Table S1). We compared random forest (RF) learning method with a classical multinomial linear regression (MLR) model in terms of performance and computation time. The computation time is 3–5 fold slower than MLR (Table S2) but the performance in terms of accuracy is moderately higher (Fig. S2). The classification strategy used in the tool is first getting votes for all 26 population groups, and generating a highest vote group as the prediction result. Then, users have options to further generate a prediction out of 5 super-population groups or 10 broader population groups as described in Section “Cross-platform benchmarking”, combining votes of groups belonging to the same super-population or broader population group, and output the group with highest vote as the prediction outcome, and a score which allows for closer scrutiny for potential admixtures.

## Supplementary information

Supplementary Information.

## Data Availability

The docker version of the tool is provided in DockerHub (*hub.docker.com/r/baudisgroup/snp2pop*). The code is available through the repository at *github.com/baudisgroup/snp2pop*.
