# Supplementary material for: Enabling population assignment from cancer genomes with SNP2pop
Source: Sci Rep. 2020 Mar 16;10:4846. doi: 10.1038/s41598-020-61854-x (PMC7075896; doi:10.1038/s41598-020-61854-x)
Supplement: Supplementary file 1 — Supplementary Information. [file 41598_2020_61854_MOESM1_ESM.pdf]

Enabling population assignment from cancer genomes with SNP2pop
Supplementary Material

Qingyao Huang and Michael Baudis

Department of Molecular Life Sciences and Swiss Institute of Bioinformatics
University of Zurich
Winterthurerstrasse 190
8057 Zurich
Switzerland
email: mbaudis@imls.uzh.ch

Suppl. Figures:

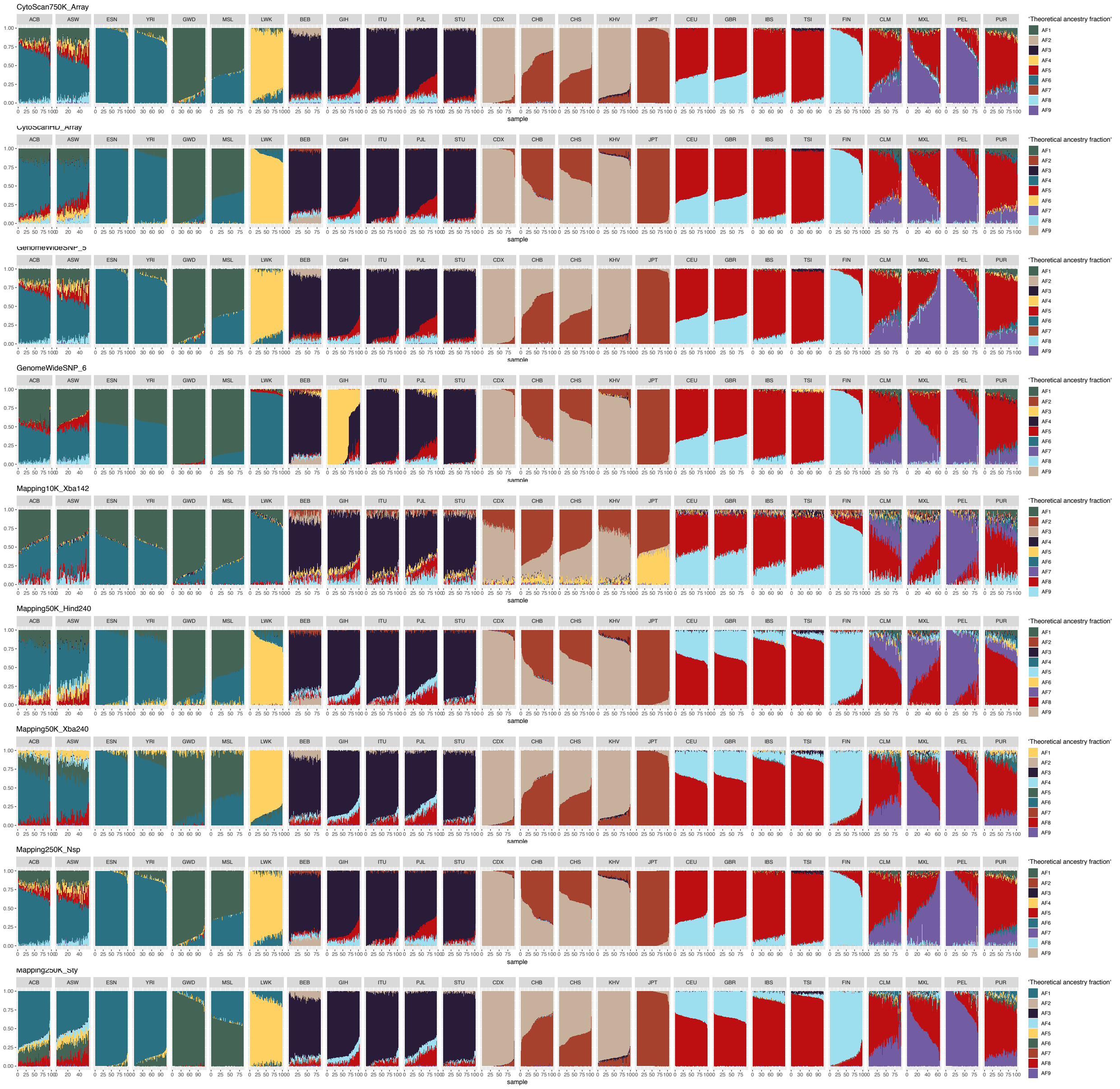

Figure S1. The fraction or contribution of theoretical ancestors (k=9) in reference individuals from 1000 Genomes Project with regard to nine SNP array platforms. The x-axis are individual samples, grouped by their respective population. Groups belonging to the same continent/superpopulation are placed neighboring to each other: AFR (1-7), SAS (8-12), EAS (13-17), EUR (18-22), AMR (23-26).

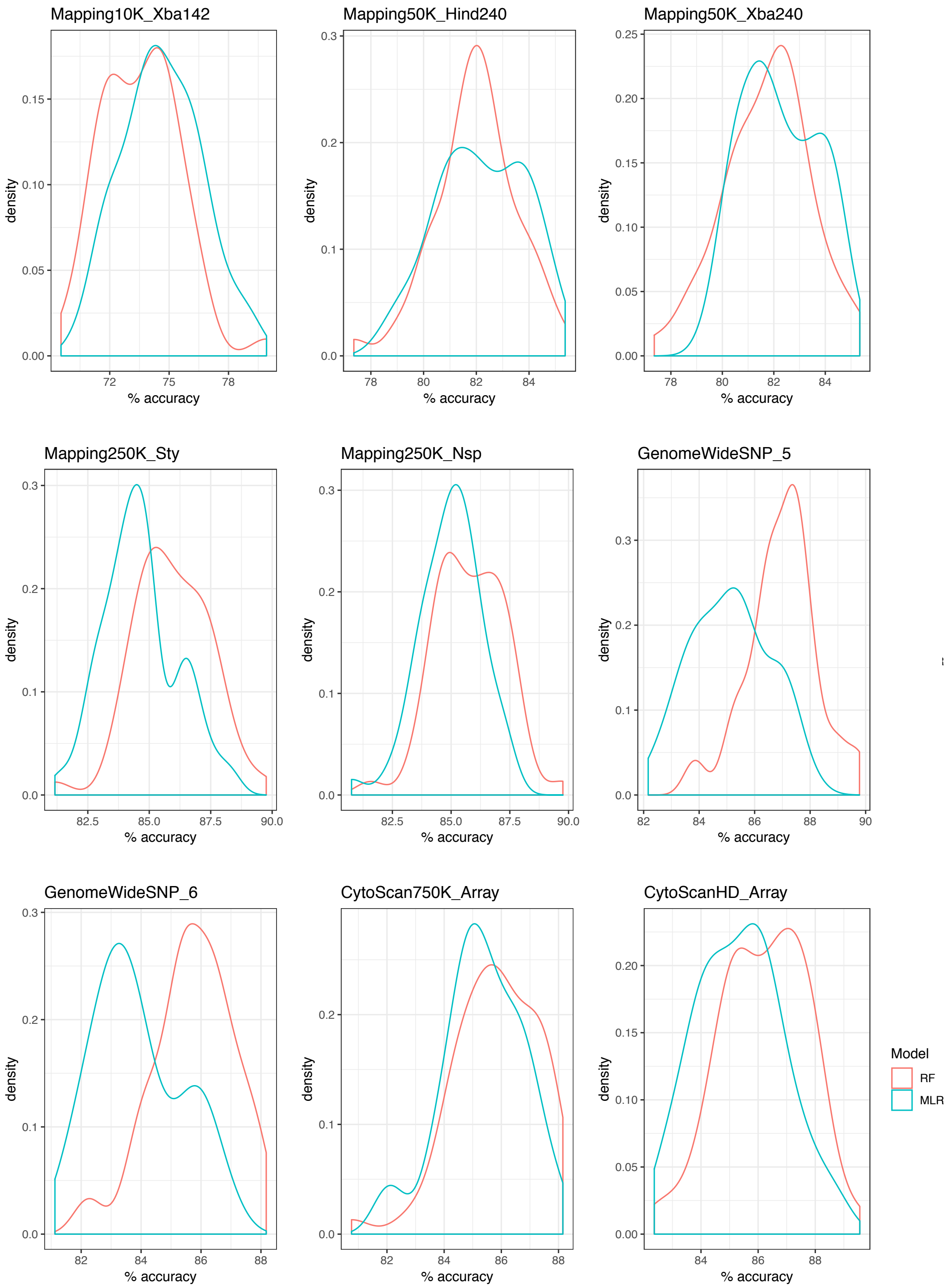

Figure S2. **Percentage of accurate prediction with multinomial linear regression (MLR) model and random forest (RF) model into 26 population groups defined in 1000 Genomes Project.**

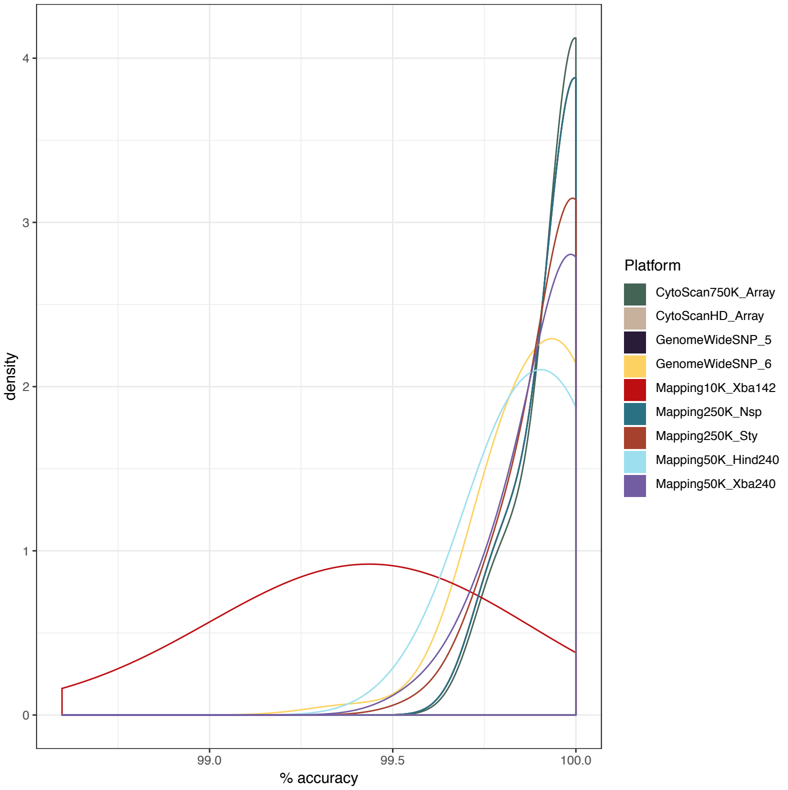

Figure S3. Percentage of accurate prediction with multinomial linear regression (MLR) model and random forest (RF) model into 10 population groups defined by the classification ambiguity between 26 groups. The frequently mixed groups are merged into one, leaving 10 out of 26 groups. See Supplementary Table S3 for the merged groups.

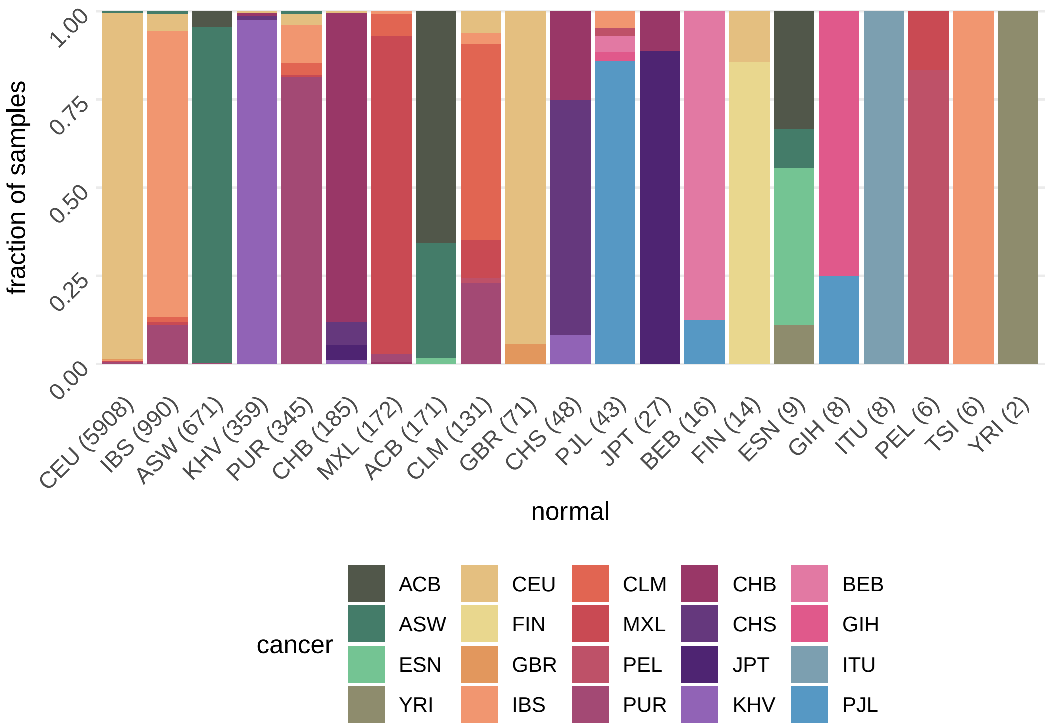

Figure S4. Agreement of assigned population groups between paired tumor and normal samples in TCGA project on the level of 26 population groups.

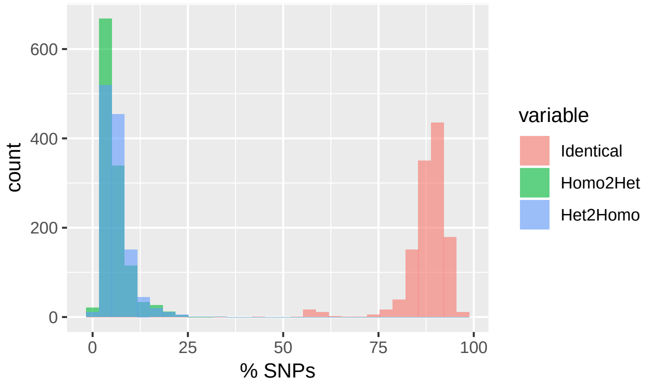

Figure S5. Percentage of SNPs in the genotyping arrays that stay identical or changed status. (Homo2Het: homozygous to heterozygous or Het2Homo: heterozygous to homozygous) between paired cancer and normal samples.

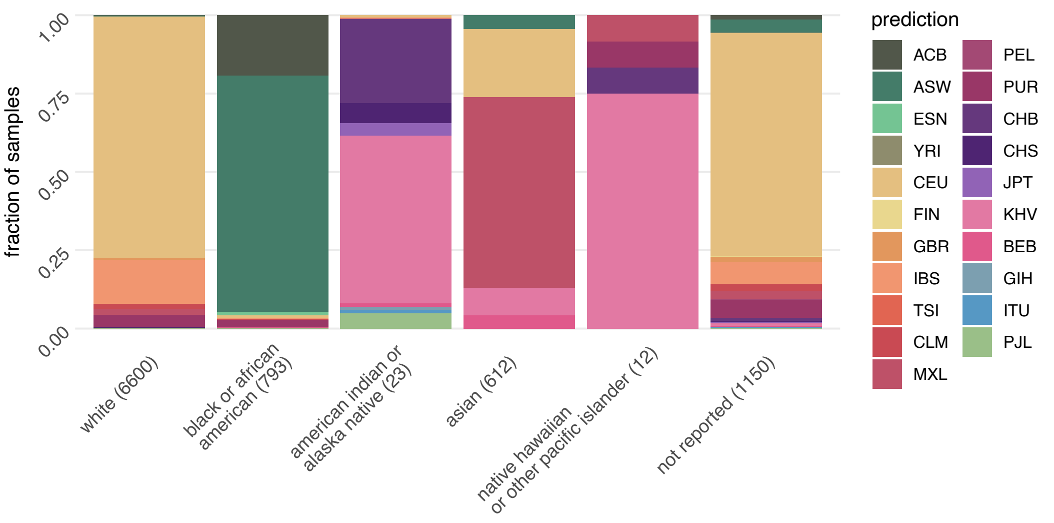

Figure S6. **The composition of assigned population groups in six "race" categories from TCGA meta-data on the level of 26 population groups.**

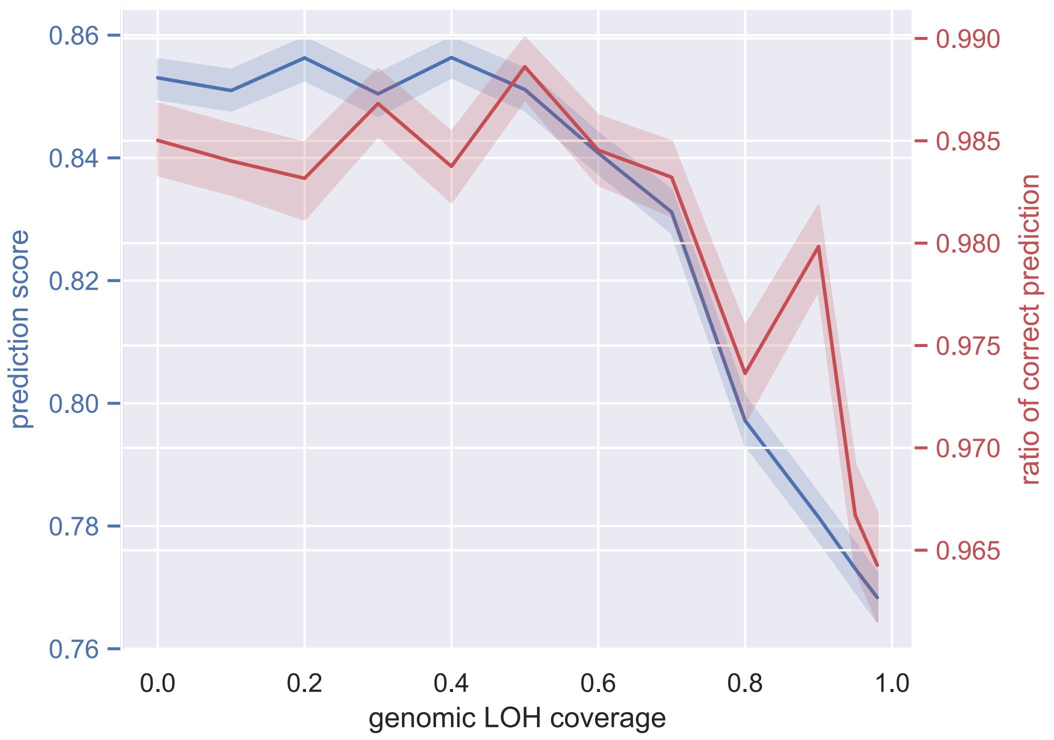

Figure S7. **The relation of correct assignment rate (blue) and confidence score (red) at different levels of LOH coverage by simulation.** In the simulation, LOH were introduced at randomly chosen 10, 20, . . . , 90, 95 and 98% of genomic SNP positions. Original data come from 1000 Genomes project where individual population groups are taken out of reference set and tested for assignment performance with simulation. LWK, FIN, JPT and GIH groups are removed for this test as they are different from other population groups in the same continental group.

Suppl. Tables: Enabling population assignment from cancer genomes with SNP2pop

Table S1. 12 IDs were removed from the reference set for the final prediction model due to high admixture background.

Sample.name	Sex	Biosample.ID	Population.code	Superpopulation.code
HG00373	female	SAME124749	FIN	EUR
HG01275	female	SAME123002	CLM	AMR
NA20299	female	SAME124381	ASW	AFR
NA20314	female	SAME125304	ASW	AFR
NA19921	female	SAME124712	ASW	AFR
HG01241	male	SAME125065	PUR	AMR
NA19625	female	SAME123655	ASW	AFR
NA19679	male	SAME122796	MXL	AMR
HG01108	female	SAME123103	PUR	AMR
HG01242	female	SAME125064	PUR	AMR
HG01438	female	SAME124971	CLM	AMR
NA20274	female	SAME123615	ASW	AFR

Table S2. Computation time per cross validation run with multinomial linear regression (MLR) model and random forest (RF) model with respect to the number of prediction output groups.

platform	No.prediction labels	MLR	RF
CytoScan750K_Array	5	1.57	13.9
CytoScanHD_Array	5	1.57	13.8
GenomeWideSNP_6	5	1.69	13.6
Mapping10K_Xba142	5	1.7	14.5
Mapping250K_Sty	5	1.59	13.9
Mapping50K_Hind240	5	1.54	14.1
CytoScan750K_Array	10	3.55	15.6
CytoScanHD_Array	10	3.48	15.5
GenomeWideSNP_6	10	3.56	15.6
Mapping10K_Xba142	10	3.96	17.1
Mapping250K_Sty	10	3.37	15.1
Mapping50K_Hind240	10	3.56	15.7
CytoScan750K_Array	26	10.2	32
CytoScanHD_Array	26	10.3	30.8
GenomeWideSNP_6	26	10.4	30.7
Mapping10K_Xba142	26	10.2	35.8
Mapping250K_Sty	26	10.3	31.9
Mapping50K_Hind240	26	10.3	33.2

Table S3. 10 groups can be distinguished from each other, based on the platform-derived SNPs. AAfr: West Africa+Caribbeans+African American; WCAfr: West Coast Africa

AAfr	South Asia	East Asia	Central Europe	South America	WCAfr	Finnish	Japan	Luhya	South Europe
ACB	BEB	CDX	CEU	CLM	GWD	FIN	JPT	LWK	IBS
ASW	GIH	CHB	GBR	MXL	MSL				TSI
ESN	ITU	CHS		PEL					
YRI	PJL	KHV		PUR					
	STU								

Table S4. Assignment concordance between paired samples in relation to assignment score range for 1145 paired cancer and normal samples from GEO.

score_range	No.sample	mismatch	error_rate
(-0.001,0.2)	527	84	0.1594
(0.2,0.4)	403	6	0.0149
(0.4,0.6)	65	1	0.0154
(0.6,0.8)	126	0	0
(0.8,1)	98	0	0

Table S5. Assignment concordance between paired samples in relation to assignment score range for 9190 paired cancer and normal samples from TCGA.

score_range	No.sample	mismatch	error_rate
(-0.001,0.2)	974	245	0.2515
(0.2,0.4)	490	17	0.0347
(0.4,0.6)	387	3	0.0078
(0.6,0.8)	1961	0	0
(0.8,1)	5378	1	2.00E-04
